# Patients with hematologic malignancies have many reasons to die during extracorporeal membrane oxygenation

**DOI:** 10.1186/s13054-014-0522-0

**Published:** 2014-09-15

**Authors:** Matthieu Schmidt, Daniel Brodie, Alain Combes

**Affiliations:** Medical-Surgical Intensive Care Unit, iCAN, Institute of Cardiometabolism and Nutrition, Hôpital de la Pitié–Salpêtrière, Assistance Publique–Hôpitaux de Paris, Paris, France

We read with great interest the article by Wohlfarth and colleagues [1] regarding the use of extracorporeal membrane oxygenation (ECMO) in 14 adult patients with hematological malignancies. We would like to highlight two main points.

First, the authors focused their report and their discussion on bleeding complications and anticoagulation management in this high-risk population. Although we concede that it is a serious concern in these patients, it is not the only one. ECMO support is associated with nosocomial infections [2,3]. Impairment of cellular immunity, cytopenia and chemotherapy (CT) may further increase the risks of infection and may dissuade clinicians from using ECMO in these patients. Developing new strategies that aim to limit nosocomial infections is crucial to improving outcomes in this population. ECMO in awake, non-intubated, spontaneously breathing patients with acute respiratory distress syndrome, to avoid mechanical ventilation and its related adverse events, is a potentially promising application [4]. Thus, in our opinion, it would also be important to provide a thorough description of nosocomial infections that might have occurred in these 14 patients.

Second, the authors reported that 5 of 14 patients initially received CT while receiving ECMO. The pharmacokinetics of many of the medications administered to patients receiving ECMO are complex [5] and, to date, there are very limited data to guide our daily practice. Therefore, clinicians must be aware that providing CT to patients receiving ECMO is a potential gamble, which risks worsening patient outcomes due to ineffective drug regimens. CT during ECMO should be restricted to those cases where postponing therapy is not an option.

Authors’ response

Philipp Wohlfarth, Thomas Staudinger and Peter Schellongowski

We thank the authors for their thoughtful considerations. Eight of the ten cases of pneumonia leading to ECMO were hospital-acquired. However, the number of proven infections during the ICU stay was low. Of 110 cultures (broncho-alveolar lavage, blood, urine, catheters, stool, pleural/pericardial effusion, lung biopsies, wound swaps, cerebrospinal fluid), only 6 were positive (catheter-related infection, n = 5; urinary tract infection, n = 1). Reactivation of cytomegalovirus or herpes simplex virus occurred in one and three patients, respectively. All patients had received broad-spectrum antibiotics, eight of them plus antifungals.

Even apart from ECMO, optimal timing, pharmacokinetics and, therefore, dosing of CT in patients with acute organ dysfunctions is largely unknown. Nevertheless, CT may be the only option if hematologic malignancies themselves cause organ dysfunction, especially in pulmonary involvement [6]. Nowadays, administration of CT is common practice in critically ill patients and affects every fourth ICU patient with hematologic malignancies in dedicated centers [7]. Importantly, CT does not negatively influence survival in large cohorts, even in cases of subsequent sepsis [7-9]. By clinical judgment, postponing CT was not an option in any of our five patients, of whom three are in complete remission after 13, 15 and 46 months.

As a survival benefit of veno-venous ECMO still has to be proven in patients with acute respiratory failure, thorough case-by-case evaluation is absolutely essential. According to our findings, the risk of nosocomial infections or CT-related issues should not lead to general exclusion of patients with hematologic malignancies from ECMO. Further research is warranted.

## Abbreviations

CT: Chemotherapy
ECMO: Extracorporeal membrane oxygenation
